# Histological assessment of cortical bone changes in diabetic rats

**DOI:** 10.1186/s13018-022-03471-0

**Published:** 2022-12-27

**Authors:** Masataka Minami, Kazuya Ikoma, Okihiro Onishi, Motoyuki Horii, Kyoko Itoh, Kenji Takahashi

**Affiliations:** 1 Department of Orthopaedics, Graduate School of Medical Science, Kyoto Prefectural University of Medicine, Kawaramachi-Hirokoji, Kamigyo-Ku, Kyoto, 602-8566 Japan; 2 Department of Pathology and Applied Neurobiology, Graduate School of Medical Science, Kyoto Prefectural University of Medicine, Kyoto, Japan

**Keywords:** Diabetes mellitus, Bone quality, Bone histomorphometry

## Abstract

**Background:**

Diabetes mellitus weakens bone strength due to deterioration of bone quality; however, the histological mechanisms are still unknown. We hypothesized that histological assessment of cortical bone would enable us to determine the cause of the bone strength reduction associated with diabetes mellitus. Our aim was to evaluate the histomorphometric changes of cortical bone associated with deterioration of intrinsic bone properties and bone quality in diabetes mellitus.

**Methods:**

We compared the outcomes of mechanical tests, bone mineral density measured using micro-computed tomography, and histological assessments, by applying Villanueva’s bone stain, to the tibial bones of 40-week-old diabetic and control male rats.

**Results:**

With respect to mechanical testing, the maximum load and energy absorption were significantly lower in the diabetic than in the control group, although fracture displacement and stiffness were not significantly different between the two groups. Bone mineral density was significantly higher in the diabetic group than in the control group. Bone histomorphometry revealed that the diabetic rats had fewer osteocytes, greater cortical porosity, and increased mineralization in cortical bone compared with the control group.

**Conclusions:**

Increased mineralization of the cortical bone with greater cortical porosity leads to a weakening of bone strength in diabetes mellitus.

## Background

Type 2 diabetes mellitus (DM) is a lifestyle-related disease that affects more than 400 million people worldwide and results in various health-related complications [1]. One of these complications is reduced bone strength, which increases the risk of fractures [2]. In fact, type 2 DM is associated with a 1.38-fold higher risk of proximal femoral fractures [3]. Notably, areal bone mineral density, measured by dual-energy X-ray absorptiometry, among patients with type 2 DM is considered to be normal or higher than normal [4]. Bone strength is determined by both bone mineral density and bone quality; therefore, the increased risk for fractures associated with type 2 DM is thought to result from a deterioration in bone quality [5, 6].

Bone quality is determined by the microcellular structure and material properties of bone [7]. The mineral density is also a major determinant of bone strength. These material properties are affected by the quality of collagen, which accounts for more than 90% of the protein content of bone [8]. A study using high-performance liquid chromatography identified an increased prevalence of abnormal collagen cross-linkages in bones among patients with DM [9]. The few studies on histological assessments of bone in DM have only included human samples obtained by biopsy or through autopsy. Recently, Hunt et al. reported that worsening glycemic control was associated with greater mineral content and narrower distributions of acid phosphate using Fourier-transform infrared (FTIR) for the samples obtained by biopsy [10]. However, the actual histological mechanism of bone quality deterioration in DM is largely unknown [11].

In this study, we focused on cortical bone as a factor for reduced bone strength. Cortical bone accounts for 65% of bone strength, contributing markedly to the strength of long bones [12]. We hypothesized that histological assessment of cortical bone would enable us to determine the cause of the bone strength reduction associated with type 2 DM.

Bone morphology can be assessed in detail by applying Villanueva’s bone stain to samples of non-decalcified bone [13]. Considering that this method involves non-decalcified bone samples and can distinguish a newly formed bone by detecting the labeling under fluorescent light, a combination of natural light and fluorescent light enables assessment of normal osteocytes, bone mineralization, and newly formed bone. Therefore, by applying Villanueva’s bone stain to samples of non-decalcified bone, we aimed to determine cortical bone changes associated with DM from a histological perspective.

## Materials and methods

### Statement of ethics

This study was carried out in strict accordance with the recommendations from the Guide for the Care and Use of Laboratory Animals of the National Institutes of Health. This research was approved by the Animal Care Committee of the Kyoto Prefectural University of Medicine, Kyoto, Japan (Protocol Number: M29-506), and performed in compliance with ARRIVE guidelines. All experiments were performed under isoflurane inhalation anesthesia, where all efforts were made to minimize animal suffering.

### Animal model

The diabetic group consisted of 40-week-old WBN/Kob rats (*N* = 16; Shimizu Laboratory Supplies Co., Ltd., Kyoto, Japan), and the control group consisted of 40-week-old Wistar/ST rats (*N* = 16; Shimizu Laboratory Supplies Co., Ltd., Kyoto, Japan). The rats were all male in order to remove the influence of sex hormones related to the menstrual cycle, such as estrogen, on measured bone outcomes. The rats were raised in an animal room, at a temperature of 23–24 °C and under a 12-h light/12-h dark cycle. All animals had free access to food and water. For bone labeling, tetracycline hydrochloride (Tc, 10 mg; Sigma-Aldrich Co., St. Louis, MO, USA) and calcein (Cl, 5 mg; Sigma-Aldrich Co., St. Louis, MO, USA) were injected subcutaneously at 10 days and 4 days, respectively, prior to euthanasia. The labeling schedule was set to a longer interval based on previous reports which demonstrated that, in cortical bone, bone formation occurs through modeling, when bone resorption does not precede bone formation [14].

### Serum testing

All animals were fasted for 10 h prior to being euthanized, but with free access to water. Prior to euthanasia, blood samples were obtained from the heart under isoflurane inhalation anesthesia. Blood urea nitrogen and creatinine levels were measured as markers of renal function. Fasting blood glucose and glycated albumin (GA) levels were measured using enzymatic methods, providing measures of blood glucose. Animals were then euthanized by intraperitoneal injection of 100 mg/kg pentobarbital. Shortly after euthanization, the right tibia was harvested from each animal. For both groups, samples from eight rats were used for micro-computed tomography (µCT) and mechanical testing, where samples from the other eight rats were used for histological assessment. For µCT and mechanical testing, soft tissues were removed and the fibulae were resected. The samples were immersed in saline solution and kept frozen until examination. For histological assessment, the samples were immediately fixed in 70% ethanol after removal and stored in a cool, dark place with light shielding.

### µCT analyses

**Fig. 1:**
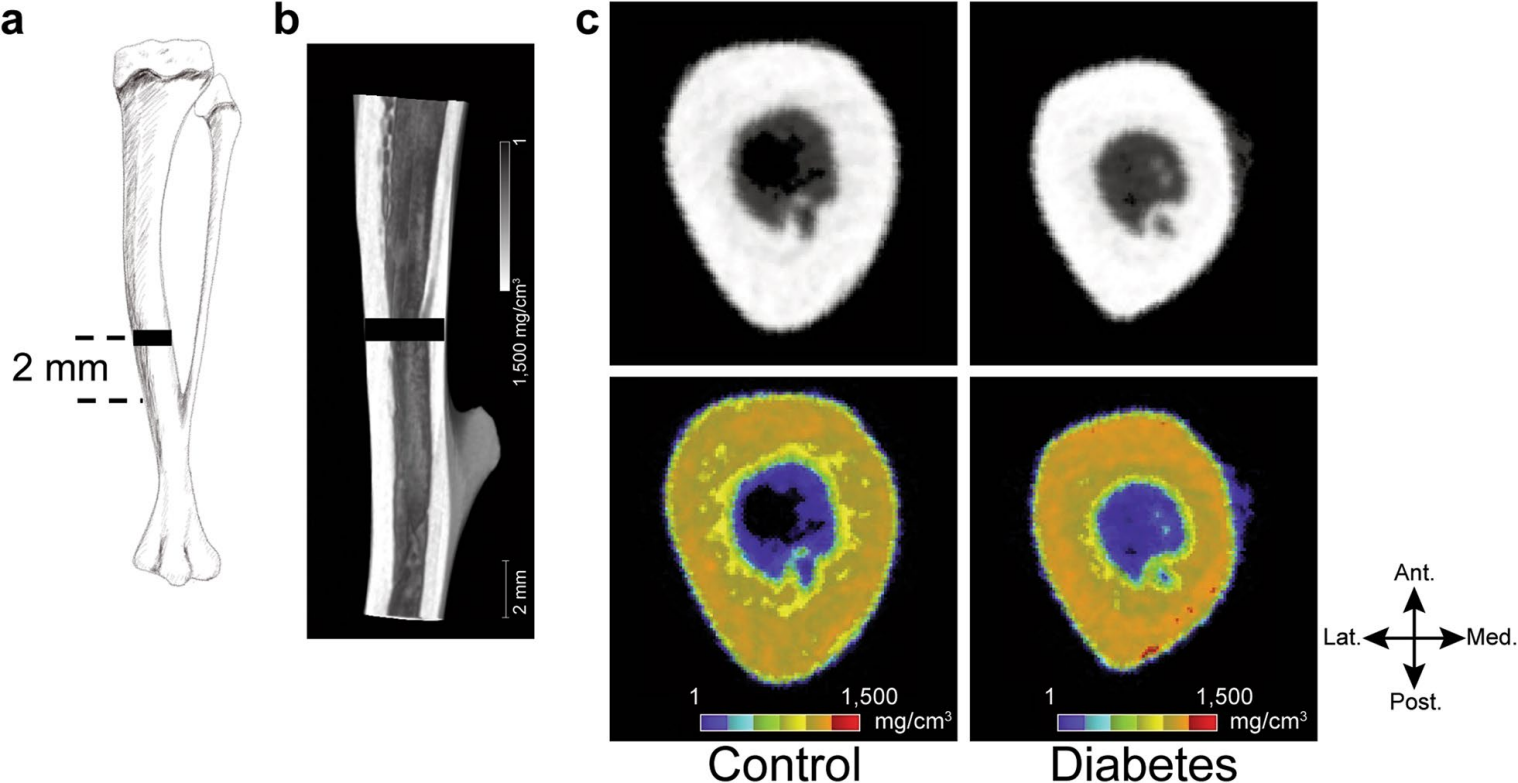
Region of interest (ROI) and representative images of micro-computed tomography (µCT). **a** The ROI is set 2 mm proximal to the tibia-fibula junction. **b** The ROI is displayed on the sagittal image of µCT. **c** Images of µCT (upper row) and calculated bone mineral density (bottom row). The mineral density of the cortical bone area is significantly higher in the diabetes group than in the control group (*p* = 0.003). *Ant.* anterior *Post.* posterior, *Med.* medial, *Lat.* lateral, *ROI* region of interest, *µCT* micro-computed tomography

Tibiae were evaluated using µCT (micro-focus 2D/3D, ScanXmate-E090S40, Comscantecno Co., Ltd., Kanagawa, Japan). Images were taken at a voltage of 60 kV and an electric current of 85 μA, with a voxel size of 37.5 μm. Bone mineral determination phantom and tibial bone samples were scanned under the same conditions, and CT intensity was converted to mg/cm^3^. Eight types of phantoms were used: 200 mg/cm^3^, 300 mg/cm^3^, 400 mg/cm^3^, 500 mg/cm^3^, 600 mg/cm^3^, 700 mg/cm^3^, 800 mg/cm^3^, and 1550 mg/cm^3^. Three-dimensional images were reconstructed using the coneCTexpress software (version 1.3; Comscantecno Co., Ltd., Kanagawa, Japan). The cortical bone area was defined by setting the threshold of CT value to ≥ 400 mg/cm^3^. The region of interest (ROI) was defined as the cross section of the entire region of cortical bone, measured 2 mm proximal to the union between the tibia and fibula (Fig. 1a, b). Cortical bone mineral density was measured in the ROI at a thickness of 200 µm, using the TRI/3D-BON software (Ratoc System Engineering Co., Ltd., Tokyo, Japan).

### Bone mechanical testing

**Fig. 2:**
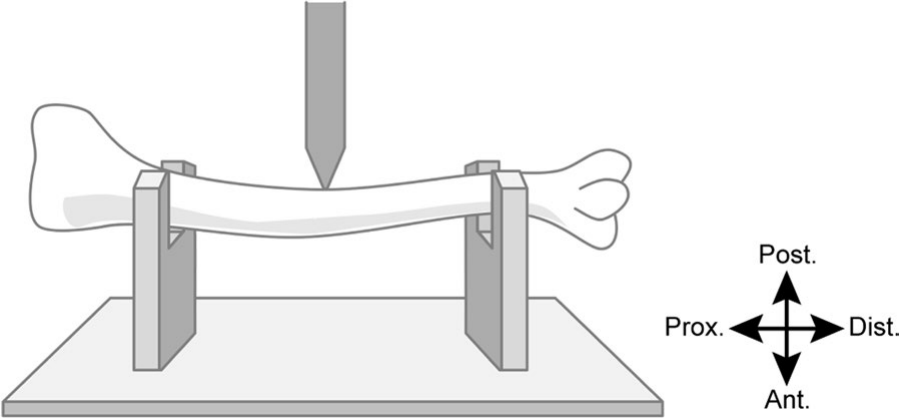
Schema of three-point bending test. The tibia was placed on two fulcra, spaced 15 mm apart, and a load was applied from the posterior toward the anterior surface of the tibia at a rate of 2 mm/min. The load was applied at the midpoint between the fulcra and was increased until the tibia fractured. *Ant.* anterior, *Post.* posterior, *Prox.* proximal, *Dist.* distal

After µCT analyses, a three-point bending test was performed using a specialized device (Maruto Co., Ltd., Tokyo, Japan) to assess bone strength. The tibia was placed on two fulcra, spaced 15 mm apart, and a load was applied from the posterior toward the anterior surface of the tibia at a rate of 2 mm/min. The load was applied at the midpoint between the fulcra and was increased until the tibia fractured (Fig. 2). The maximum load (N), fracture displacement (mm), stiffness (N/mm), and energy absorption (N mm) were quantified. Since maximum load and fracture displacement need to be adjusted for bone geometry, they were normalized by dividing by the radius of the bone calculated by µCT, and the results were compared between the two groups.

### Bone histomorphometry

The resected right tibiae were fixed in 70% ethanol and treated with Villanueva osteochrome bone stain. Samples were embedded in methyl methacrylate, and cross-sectional slices were prepared at the ROI, 2 mm proximal to the union between the tibia and fibula. To calculate morphometric parameters, a single experienced examiner conducted the analyses under natural and fluorescent light (described below), in accordance with Parfitt’s method [15].

#### Histomorphometric data

All histomorphometric data were described as ASBMR nomenclature [16]. Overall, all histomorphometric data were described according to the American Society for Bone and Mineral Research (ASBMR) nomenclature. First, cortical area (Ct.Ar) and marrow area (Ma.Ar) were measured under natural light, at a 100× magnification. These measurements were used to calculate the Ma.Ar/Ct.Ar ratio.

#### Periosteum and endosteum

**Fig. 3:**
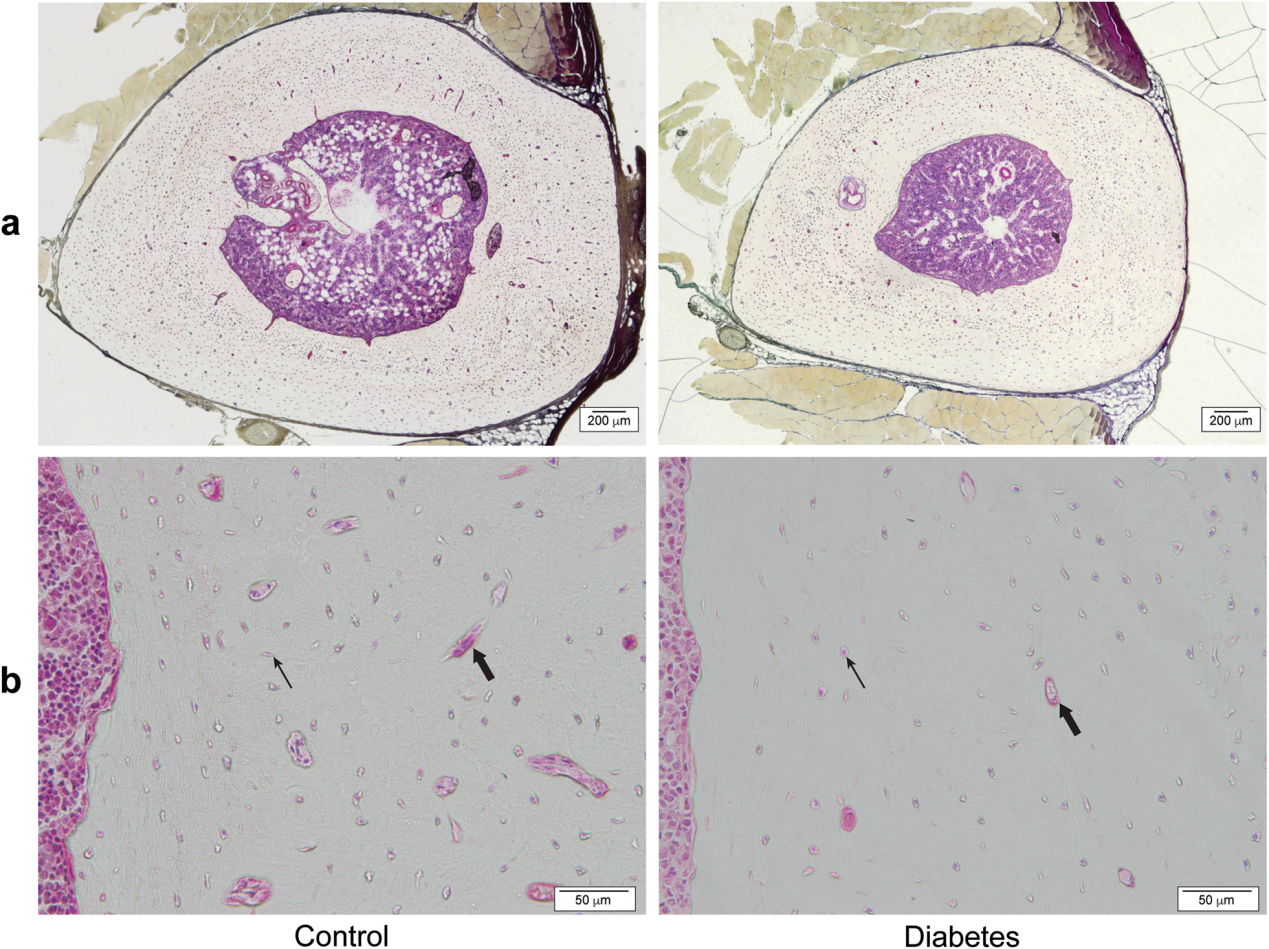
Histological tibial transections observed under natural light for the control and diabetes groups. **a** Low-power field, **b** High-power field. The number of osteocytes (thin arrow) by bone volume is significantly smaller in the diabetic group than in the control group (*p* = 0.03). Cortical porosity (bold arrow) is significantly lower in the diabetic group than in the control group

**Fig. 4:**
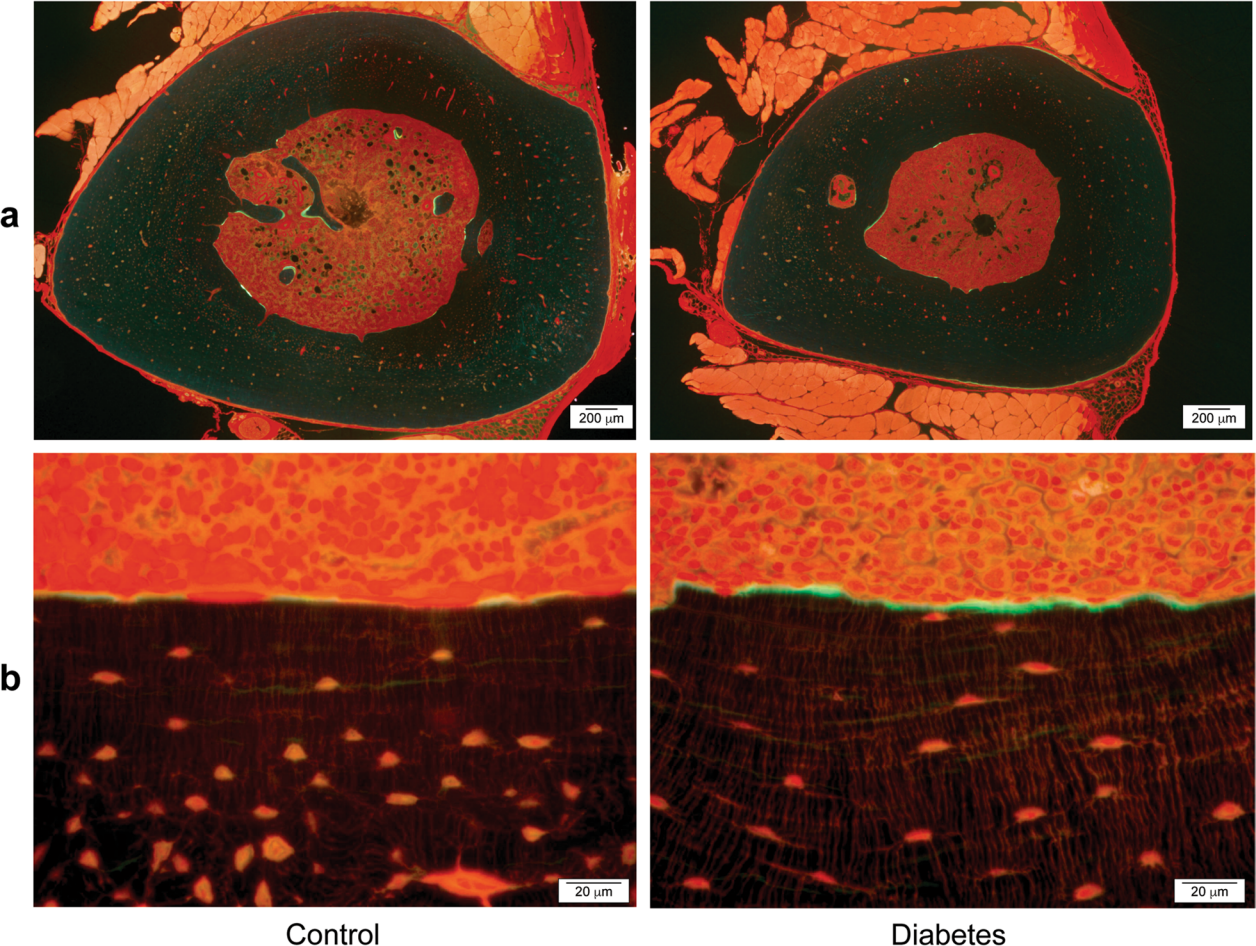
Histological tibial transections observed under fluorescent light for the control and diabetes groups. **a** Low-power field, **b** High-power field. The total length of mineral deposit; the fluorescently marked green line is significantly greater in the diabetic group than in the control group (*p* = 0.003)

The osteoid surface (OS) in periosteum, eroded surface (ES) in periosteum, and quiescent surfaces (QS) were distinguished in the endosteum and the periosteum under natural light, at a 200× magnification. The percentage of OS, ES, and QS in the endosteum and periosteum were calculated (Fig. 3). Labeled surfaces were measured under fluorescent light, and the percentage of labeled surfaces in the periosteum and endosteum were calculated (Fig. 4). The labeled surface-to-bone surface (MS/BS) ratio and the mineral apposition rate (MAR) were measured by distinguishing the single-labeled surface (sLs) and double-labeled surface (dLs), which could be recognized from the tetracycline and calcein labeling under fluorescent light. The sLs and dLs were measured around the entire periosteum and endosteum. The bone formation rate (BFR) was calculated using the following formula: BFR/BS = (sLS + 2 × dLS) × MAR/BS. Of the labeled sites in the endosteum, excessively stained sites in which osteoblasts were not observed were determined to be sites of mineral deposit. Mineral deposit was calculated separately from other mineralization.

#### Cortical porosity

The void area, which includes Volkmann’s canals and blood vessels, throughout the cortical bone was calculated under natural light at a 200× magnification (Fig. 3). We then calculated the cortical porosity (Co.P) within the cortical bone.

#### Osteocytes

The osteocyte number (N.Ot) was calculated under natural light at a 400× magnification in six visual fields in each sample in the site adjacent to the endosteum, on the medial side of the tibia. We calculated the N.Ot by BV, expressed as N.Ot/BV (Fig. 3).

### Statistical analyses

Serum tests, µCT, bone mechanical tests, and bone histomorphometric parameters were compared between the diabetic and the control groups using Student’s t test for parametric parameters and the Mann–Whitney *U* test for nonparametric parameters. Parametric values are reported as the mean ± standard deviation, with nonparametric values reported as the median and range. All statistical analyses were performed using SPSS Statistics (version 25.0; IBM Corporation, Armonk, NY, USA). A *p* value < 0.05 was considered statistically significant.

## Results

### Serum testing

In the diabetic group and in the control group, the fasting blood glucose was 248 ± 53 mg/dL and 204 ± 33 mg/dL, respectively, with GA values of 2.8 ± 1.2% and 1.7 ± 0.6%, respectively. Both values were significantly higher in the diabetic than in the control group (for the fasting blood glucose: *p* = 0.01, for GA: *p* = 0.01; Table 1). The blood urea nitrogen levels in the diabetic and control groups were 16.4 ± 2.5 mg/dL and 16.1 ± 4.6 mg/dL, respectively (*p* = 0.80). However, the creatinine level was significantly higher in the control (0.36 ± 0.09 mg/dL) than in the diabetic (0.27 ± 0.02 mg/dL) group (*p* = 0.001).

**Table 1:** Results of serum testing for control and diabetic rats

	Control	Diabetic	*p*-value
Fasting blood glucose (mg/dL)	204 ± 33	248 ± 53	0.01
GA (%)	1.7 ± 0.6	2.8 ± 1.2	0.01
BUN (mg/dL)	16.1 ± 4.6	16.4 ± 2.5	0.80
Cre (mg/dL)	0.36 ± 0.09	0.27 ± 0.02	0.001

Values are expressed as means ± standard deviations

*GA* glycated albumin, *BUN* blood urea nitrogen, *Cre* creatinine

### Micro-computed tomography (µCT) analyses

Cortical mineral density, measured at the ROI, was 1339 ± 15 mg/cm^3^ in the diabetic group and 1313 ± 12 mg/cm^3^ in the control group, indicative of a significantly higher bone mineral density for the diabetic than control group (*p* = 0.003; Fig. 1c). The location of the ROI and a representative image are shown in Fig. 1a, b.

### Bone mechanical testing

For one sample in the control group, a uniform load could not be applied; this sample was excluded from mechanical testing analysis. The maximum load in the diabetic group was 111 ± 9 N, compared to 133 ± 12 N in the control group. After adjustment, the maximum load was significantly lower in the diabetic than control group (*p* = 0.04; Table 2). Energy absorption in the diabetic group was 66 ± 8 N mm, compared to 92 ± 21 N mm in the control group, with energy absorption being significantly lower in the diabetic group (*p* = 0.02). Fracture displacement in the diabetic group was 0.8 ± 0.1 mm, compared to 1.0 ± 0.2 mm in the control group; this between-group difference was not significant after adjustment (*p* = 0.28). Stiffness was also not significantly different between the diabetic (355 ± 36 N/mm) and control (352 ± 20 N/mm) group (*p* = 0.87).

**Table 2:** Results of bone mechanical testing for control and diabetic rats

	Control	Diabetic	*p*-value
Maximum load (N)	133 ± 12	111 ± 9	0.04
Fracture displacement (mm)	1.0 ± 0.2	0.8 ± 0.1	0.28
Stiffness (N/mm)	352 ± 20	355 ± 36	0.87
Energy absorption (N mm)	92 ± 21	66 ± 8	0.02

Values are expressed as means ± standard deviations

### Bone histomorphometry

#### Overall

Ct.Ar was significantly smaller in the diabetic than in the control group (*p* < 0.001; Table 3). The Ma.Ar was significantly smaller in the diabetic than in the control group (*p* < 0.001; Fig. 3a). The Ma.Ar/Ct.Ar ratio was significantly smaller in the diabetic than in the control group (*p* = 0.01).

**Table 3:** Results of bone histomorphometry for control and diabetic rats

	Control	Diabetic	*p*-value
Ct.Ar (mm^2^)	5.2 ± 0.3	4.3 ± 0.2	< 0.001
Ma.Ar (mm^2^)	1.5 ± 0.2	1.0 ± 0.1	< 0.001
Ma.Ar/Ct.Ar (%)	23.2 (17.7–25.0)	18.5 (18.0–20.4)	0.01
OS (%)	28.5 (0.0–75.3)	15.7 (0.0–30.1)	0.28
ES (%)	5.9 (3.3–11.7)	6.5 (4.0–15.3)	0.44
MS/BS (Ps) (%)	9.6 (0.0–46.8)	10.0 (0.0–23.9)	0.88
MS/BS (Es) (%)	1.0 (0.0–7.3)	0.0 (0.0–8.2)	0.33
MS/BS (Ps + Es) (%)	6.8 (0.0–31.2)	7.2 (0.0–19.2)	0.65
MAR (µm/day)	0.4 ± 0.4	0.5 ± 0.4	0.72
BFR/BS (µm^3^/µm^2^/day)	0.12 ± 0.15	0.08 ± 0.07	1.00
Ct.P (%)	0.7 (0.6–0.9)	0.6 (0.4–0.6)	< 0.001
N.Ot/BV	776 (523–1083)	601 (518–870)	0.03

Values are expressed as means ± standard deviations for parametric parameters and as medians and ranges for nonparametric parameters

*Ct.Ar* cortical area, *Ma.Ar* marrow area, *OS* osteoid surface in periosteum, *ES* eroded surface in periosteum, *MS* mineralizing surface, *BS* bone surface, *PS* periosteal, *ES* endosteal, *MAR* mineral apposition rate, *BFR* bone formation rate, *Ct.P* cortical porosity, *N.Ot* osteocyte number, *BV* bone volume

#### Periosteum and endosteum

The average lengths of the OS and QS in the periostea of the tibiae were 1.2 mm (15.7% [0.0–31.0]) and 7.5 mm (84.3% [69.0–100.0]), respectively, in the diabetic group, compared to 3.3 mm (28.5% [0.0–75.3]) and 6.4 mm (71.5% [24.7–100.0]), respectively, in the control group (Fig. 4a). No ESs were noted in the periostea of both the groups. Thus, the percentage of OS in the periosteum was not significantly different between the two groups (*p* = 0.28). The average lengths of the ES and QS in the endostea were 0.3 mm (6.5% [4.0–15.3]) and 3.5 mm (93.5% [84.7–96.0]), respectively, in the diabetic group, compared to 0.3 mm (5.9% [3.3–11.7]) and 4.7 mm (94.1% [88.3–96.7]), respectively, in the control group. Thus, the percentage of ES in the endosteum was not significantly different between the two groups (*p* = 0.44). MS (MS/BS ratio) in the periostea was not significantly different between the two groups: 10.0% (0.0–23.9) in the diabetic group and 9.6% (0.0–46.8) in the control group (*p* = 0.88). Similarly, the MS/BS ratio in the endostea was not significantly different between the diabetic (0.0% [0.0–8.2]) and control (1.0% [0.0–7.3]) group (*p* = 0.33). The MAR (µm/day) in the periostea was not significantly different between the two groups (0.5 ± 0.4 µm/day, diabetic group; 0.4 ± 0.4 µm/day, control group; *p* = 0.72). BFR/BS (µm^3^/µm^2^/day) obtained from these results was also not significantly different between the two groups (0.08 ± 0.07 µm^3^/µm^2^/day, diabetic group; 0.12 ± 0.15 µm^3^/µm^2^/day, control group; *p* = 1.00). The total length of the prominent fluorescently labeled sites in the endostea, wherein osteoblasts were not observed (i.e., zones of mineral deposit), was significantly greater in the diabetic (747 ± 700 µm; 14%) than in the control (211 ± 63 µm; 4%) group (*p* = 0.003; Fig. 4b).

#### Cortical porosity

Co.P was significantly lower in the diabetic (0.6% [0.4–0.6]) than in the control (0.7% [0.6–0.9]) group (*p* < 0.001; Fig. 3b, bold arrow).

#### Osteocytes

The measured BV in the diabetic and the control group was 0.29 ± 0.01 mm^2^ and 0.29 ± 0.02 mm^2^, respectively, with this between-group difference not being significant (*p* = 0.65). The number of osteocytes by BV (N.Ot/BV) in the diabetic group (601 [518–870]) was significantly less than in the control group (776 [523–1083]) (*p* = 0.03, Fig. 3b, thin arrow).

## Discussion

While the rat model of DM in the present study demonstrated bone fragility on mechanical testing, µCT analysis revealed that the cortical bone mineral density had actually increased. Bone histomorphometry revealed a decrease in osteocyte count and an increase in mineral deposit within the cortical bone of the harvested right tibiae. To our knowledge, this is the first study to demonstrate an increase in mineral deposit in the cortical bone of diabetic rats.

The WBN/Kob rat is a model of DM resulting from pancreatic exocrine hypofunction, which has been used in the past to assess cortical bone [17]. We considered that this model was valid in our study, exhibiting a significant elevation in the level of blood glucose and GA. We confirmed that neither blood urea nitrogen nor creatinine levels were elevated, indicating that renal function was not diminished and, as such, identified changes in the bones in our study were not dependent on diminished renal function. In DM, insulin deficiency causes inhibition of osteoblast activity, which can reduce (or even inhibit) cortical bone modeling. This could lead to reductions in the overall bone size and numbers of osteocytes [18].

Bone mineral density, measured by µCT, was increased in the diabetic rats. Considering the fact that cortical porosity was significantly lower in the diabetic rats compared to the control rats, according to histomorphometric evaluation, this may have resulted in a relative increase in bone mineral density. This is consistent with previous studies that have also shown an increase in bone mineral density in patients with DM [2, 3]. Subsequently, cortical porosity may be relatively decreased, which is consistent with the results of our study.

On mechanical testing, the tibiae in the diabetic group sustained smaller maximum loads and reduced bone energy absorption than the control group, but without a lower stiffness. Thus, the tibiae in the diabetic rats were hard, but weak. This result is consistent with previous studies that reported an increase in abnormal collagen cross-linking associated with DM [9]. Accumulation of advanced glycosylation end products increases abnormal collagen cross-linking, which in turn results in large numbers of bonds between collagen molecules. This phenomenon may have reduced collagen energy absorption and, therefore, bone energy absorption in the subjects in this study.

Bone histomorphometry revealed a significantly greater volume of mineral deposit in the diabetic than in the control group. In general, ossification is broadly divided into the following processes: mineralization, which is driven by osteoblasts; endochondral ossification, which is driven by chondrocytes; and intramembranous ossification, which is driven by mesenchymal cells [19]. The mineral deposit observed in the diabetic group in our study was considered not to fall under any of the above processes. Specifically, mineral deposit was significantly greater in the diabetic than in the control group, creating the appearance that bone mineral density was preserved. The number of osteocytes was decreased in the diabetic compared to the control group, which may be due to the apoptosis of osteocytes by secondary mineralization. It is speculated that the decrease in the number of osteocytes may have reduced the amount of matrix, such as collagen, which should have been produced by the osteocytes. In fact, this mineral deposit was due to abnormal mineralization, possibly resulting in reduced bone absorption and bone strength. Hunt et al. reported that mineral crystallinity was observed in patients with diabetes by FTIR [10]. This mineral crystallinity may be related to the mineral deposit found in this study. Thus, abnormal mineral deposit may contribute to the reduced bone strength associated with DM.

A strength of our study was the use of an animal model of DM, which allowed us to conduct invasive mechanical testing and histological assessments. Specifically, considering that we obtained a whole bone specimen, we were able to prove bone fragility by mechanical testing and examine the relationship between this and histological evaluation in the same specimen, which has never been reported before. The limitations of our study must also be acknowledged. Specifically, identified cortical bone changes in our animal models may not occur in humans with DM. In addition, the use of individual samples limits the interpretation between histological changes and mechanical testing results. Additionally, we cannot rule out the possibility that partial volume effects may have occurred in the measurement of cortical bone BMD by µCT and may have affected the results.

## Conclusions

In conclusion, by applying Villanueva’s bone stain, we assessed cortical bone changes in a rat model of DM from a histological perspective. A reduced number of osteocytes and increased mineral deposit were observed in the diabetic group compared to the control group. Abnormal mineral deposit may lead to a deterioration of intrinsic bone properties in DM.

## Data Availability

The datasets analyzed during the current study are available from the corresponding author on reasonable request.
